# The Medicine of Work

**Published:** 1888-02-11

**Authors:** 


### THE DOCTOR'S PULPIT.

### THE MEDICINE OF WORK.

ALL the world's a school, and all the men and women merely scholars—scholars, not in the University acceptation, but in the dame-school sense—in which the biggest dunce is still a *scholar*, though a bad one. "At thirty man suspects himself a fool; knows it at forty, and reforms his plan." By common consent Young was an oracle when he gave such brief expression to so unanimous a conviction. The art of living seems as simple as a rule-of-three sum to him who has had no experience at all on the one hand, and to him, on the other, who has accepted and learnt his rules and made himself master of them by practice, it really is so. The doubting, inquiring, dissatisfied, and dreamy men and women find the world and all its cares and business unendurable.

Some people suppose the doctor has no interest in mankind except such as appertains to broken legs, colics, and other troubles of the material organism. Alas! poor doctor! if these are to be the measure of his thought and work. Pills and powders are very well in their proper place, and splints and bandages indispensable; but we submit that a mind constantly pastured on such herbage is not likely to be either well developed or exceedingly nimble at an intellectual fence. There is a limit to the mental and moral possibilities of pills, and to the cultural efficacy of even the most ingenious methods of roller bandaging. These are the details, the all-important details, of the doctor's art; but he who knows nothing except details lacks the prime essential for making these effective—namely, a comprehensive and critical intelligence.

What a superstitious and uncritical world it is! It has been accustomed from its infancy to associate its "medicine men" with pills, powders, and mixtures; and to pills, powders, and mixtures, therefore, it is resolved they shall confine themselves, on pain of being banned with "unorthodoxy" and mulcted in that most tender part, the pocket. But even a worm will turn; and the doctor, especially the nineteenth-century doctor, sometimes insists on a temporary emancipation from the thraldom of the pill-box, and resolves to take as wide a survey of men and things as his own eyes and the highest intellectual mountain he can reach the summit of will permit. At such a time of temporary liberty he sits down calmly, and shaking himself free, by an effort, from the fetters and spectacles of routine and custom, looks round from his mountain-height on the world which expands before his view.

What does the emancipated man of physic look upon? A strange medley of a world: a world of workers and idlers, of seers and sightless, of men in mad haste and men in sober locomotion, of men full of exuberant life and energy, and men in grievous pain and helplessness. He sees a world that needs all kinds of practitioners of the healing art: not merely those who can prescribe a pill for the stomach and those who can attend to an injured limb, but those whose skill extends to sins and sorrows and heartbreaks. If he look long enough from his vantage point he will see that almost every kind of medicine is useful in its turn for some or other of the world's various diseases, but he will finally discover that there is one remedy which more than any other is a sovereign balm for an innumerable number of human ills. That remedy is honest daily work.

It does not seem difficult to discover that Nature intended human creatures to work every day. To find out her real purpose we have but to go far enough back in the history of every family of man, or to count the heads of the present generation. Where there is in civilised communities one person who has resources sufficient for an idle life, there are probably five hundred who must work or starve. Among uncivilised races the proportion of those who can afford to be idle is probably much smaller. The wild creatures, which seem to have not a single care in life, are under the daily necessity of doing a good deal of hunting or fighting, or both, as their well-developed muscles or teeth amply prove. The chase or the conflict seems to constitute the chief pleasure of the fiercer animals as the cropping of the tender grass probably does that of those which are less sanguinary. But even those grass-eating creatures which do no work for man are compelled to lives of constant industry. It takes a horse or a sheep hours of energetic nibbling to satisfy the demands of a capacious stomach and to maintain the energy of the body. At the change of the seasons long fatiguing journeys must often be undertaken in search of "fresh fields"; and a flagging courage is rewarded by a "lean and hungry" experience.

It is well sometimes for a man to separate himself from his daily surroundings and to look abroad with the eyes of a reasoning being. When we understand what is the plain and evident necessity of existence for the occupants of our own particular planet we are all the more ready to say to ourselves in a convinced and willing spirit, "It is of no use to quarrel with the inevitable; that which must be will be, and there is an end of the matter." But if a man be compelled to a certain task whether he will or no there are two ways of approaching it. He may put himself into a sulky mood, and do exactly as much as the sternest necessity compels and no more. Or he may think within himself, This has got to be done; I will take off my coat, set to work with a will, and do it as promptly and as efficiently as it is in my power to do it. The former may be called the *poison* of work, the latter the *medicine*. Work done cheerfully, reasonably, and constantly will generally keep a well man healthy and will often make an ill man well. It is not of prime importance what kind of work it be, but it should if possible combine outdoor with indoor labour and the physical with the intellectual. Work of any kind done intelligently and conscientiously developes both the mind and the conscience. Among artisans and farm labourers it is practically invariable that the best workman is the most intelligent and morally worthy. The same rule obtains in what are called the learned professions. It will be found too, almost universally, that those who are the most diligent and efficient among all classes of industries are the most healthy, the most cheerful, and the longest lived. There is a wonderful medicinal potency in cheerful purpose and daily work.

Every preacher who is worth his salt does his utmost to make his sermons practical, and to give them personal application. We venture, therefore, to say, in the plainest possible manner, that there are vast numbers of both sexes whose only diseases are aimlessness and indolence. For these diseases rhubarb and magnesia are contra-indicated, and quinine is of no avail. All the professors of all the "pathies" may be consulted in turn, and all will be equally powerless to cure. If you must deal with the nettle it is safest to grasp it firmly; if the bull cannot be avoided it is best to take him by the horns. The facts must be recognised, or nothing effectual can be done. Give daily work at least a trial, and let the trial be of sufficient duration. If that were done there cannot be the least doubt that many who now have neither health nor happiness would soon find themselves in the full enjoyment of both.

